# Mitigating misinformation about the COVID-19 infodemic on social media: A conceptual framework

**DOI:** 10.4102/jamba.v15i1.1416

**Published:** 2023-05-30

**Authors:** Sivile Manene, Charity Hove, Liezel Cilliers

**Affiliations:** 1 Department of Information Systems, Faculty of Management and Commerce, University of Fort Hare, East London, South Africa

**Keywords:** infodemic, misinformation, COVID-19, social media, activity theory

## Abstract

During coronavirus disease 2019 (COVID-19), the term ‘infodemic’ was used to depict the abundance of information about COVID-19 on social media that may overwhelm users, as well as misinformation about the virus because of the lack of authentication of information posted on social media. Both the World Health Organization and United Nations have warned that infodemics can become a severe threat to health care if misinformation on social media is not addressed in a timely manner. The objective of this study was to develop a conceptual framework that can be used to mitigate misinformation about the COVID-19 infodemic on social media. A structured literature review of purposively sampled scholarly publications from academic databases was conducted. The inclusion criteria chosen were scholarly papers that investigated infodemics on social media during the COVID-19 pandemic in the past 4 years, which were analysed using thematic and content analysis. The conceptual framework used Activity Theory as the theoretical foundation. The framework identifies a set of strategies and activities for both social media platforms and users to mitigate misinformation on social media during a pandemic. This study, therefore, recommends that stakeholders utilise the developed framework on social media to reduce the spread of misinformation.

**Contribution:**

Based on the literature review, there are negative health outcomes during a social media infodemic because of the spread of misinformation on social media. The study concluded that by implementing a set of strategies and activities identified through the framework, health information can be managed on social media to improve health outcomes.

## Introduction

Coronavirus disease 2019 (COVID-19) originated in Wuhan, China, and was declared a pandemic by the World Health Organization (WHO) on 11 March 2020. According to the National Institute for Communicable Diseases (2022), as of May 2022, there were over 3 million confirmed cases and over 100 000 deaths in South Africa associated with COVID-19. The South African Government implemented a national lockdown in March 2022 to prevent the disease’s spread and not overwhelm healthcare resources. During the lockdown, South Africans stayed at home but still needed to access information regarding the virus, prevention methods, treatments and the government response. Social media became a popular communication channel for searching about COVID-19-related information. Before the COVID-19 pandemic, social media usage in South Africa stood at 21.5 million users and was projected to increase to 28 million users by 2022 (Kemp 2020; Statista 2022). To effectively communicate the COVID-19 restrictions and guidelines, the South African National Government’s Department of Communications issued the ‘Government Communication Strategy on COVID-19’. The report detailed the use of mass media platforms, that is, television and radio, and social media platforms to communicate with the public. These methods of communication became pivotal in South Africa’s efforts to effectively communicate COVID-19 preventative measures (Republic of South Africa 2020).

An infodemic refers to the overabundance of accurate and inaccurate information during a pandemic on social media, resulting in citizens’ distrust of the public health system (World Health Organization 2020). Tran et al. (2020) reported that properly regulated health information is essential because it impacts health personnel and community workers on the front lines of the pandemic. The increase in the popularity of social media to search for health information because of the lockdown in South Africa meant that social platforms became a catalyst for the current COVID-19 infodemic.

Social media does not regulate health information posted, so there is no way of knowing if the source(s) is reliable, the information is accurate, and the originator’s intent when posting the information (Cinelli et al. 2020). Misinformation is defined as false information that is spread with no ill intention from the source (Santos-D’Amorim & Fernandes De Oliveira Miranda 2020). According to Mian and Khan (2020), most misinformation during the COVID-19 pandemic emerged from social media accounts. The information posted on social media is not validated and can be spread worldwide in seconds. Health authorities also find it challenging to counteract misinformation as it is impossible to know who received the information. In addition, misinformation is more likely to be shared on social media as the posts tend to be more sensationalist (Santos-D’Amorim & Fernandes De Oliveira Miranda 2020). According to Vosoughi, Roy and Aral (2018), there is a 70% likelihood that false or misinformation will be shared before factual information on social media.

Tasnim, Hossain and Mazumder (2020) highlighted that misinformation promotes unsafe health practices, which increases the spread of COVID-19. Misinformation also harms public health as the information undermines the Government or health institutions’ efforts to fight the pandemic, which leads to mistrust among the public because of the negative perception misinformation creates. Therefore, given the impact misinformation has on COVID-19 prevention from a public health perspective, it is therefore crucial that measures are put in place to mitigate misinformation on social media. The study aims to develop a conceptual framework that can be used to mitigate misinformation about COVID-19 on social media platforms during an infodemic.

The following sections of this paper will include a literature review that will further address the given research problems, as well as a detailed explanation of the theoretical perspective, research methodology and discussion of the findings. The paper will further conclude by detailing the limitations and additional recommendations for future research based on the findings of the research conducted throughout this study.

## Literature review

During the COVID-19 pandemic, health organisations relied on social media platforms to inform people about COVID-19. There are many advantages of using social media in healthcare, such as cost-effectiveness, high social engagement with health promotion activities and the broad reach of social media. The disadvantages of using social media to distribute health information include lack of legislative jurisdiction because of social platforms being internet-based, which means that the social media company and user are in different countries, and the prevalence of false information because of the lack of verification mechanism for health information (Clark 2020). Furthermore, social media algorithms allow users and content creators to distribute content at fast rates, thus significantly increasing the spread of COVID-19-related information without assessing the validity of the information (Gisondi et al. 2022). Social media provides ‘echo chambers’ where citizens can voice their beliefs. The problem is that social media has created an environment where the most popular or shared posts can dominate the entire discussion, even if the information is scientifically inaccurate (Grewal, Dourado & Ryerson 2021).

Stakeholders that spread misinformation on social media include patients and the general public, healthcare professionals, government organisations or public health entities and social media platforms and regulators (Grewal et al. 2021). Myths can be dangerous to the general public; for example, drinking alcohol will kill the COVID-19 virus. This myth resulted in the hospitalisation of 5876 people and the deaths of over 800 people in Iran because of methanol poisoning (De Moura et al. 2021). Misinformation also impacts healthcare professionals and health resources. Monostra (2020) found the myths about the lack of efficacy of masks that children are immune from COVID-19 and herd immunity as reasons why COVID-19 infections increased worldwide.

Social media platforms have taken steps to mitigate the spread of COVID-19 misinformation by implementing policies such as Twitter’s recent ‘COVID-19 misleading information policy, which prohibits users from using the platform’s services to share false or misleading information about COVID-19’ (Twitter 2022). Olan et al. (2022) reported that societal acceptance of information depends on the verification features available on social media platforms. The lack of verification further highlights the need for social media platforms to introduce mechanisms that fact-check and verify the information to reduce the spread of misinformation on social media platforms (Olan et al. 2022).

Cuello-Garcia, Perez-Gaxiola and Van Amelsvoort (2020:199) derived a list of strategies to counteract misinformation on social media platforms. These strategies consisted of the following: scientists should actively participate on social media; there should be assistance and advocation for expert fact-checking; health experts and scientists should publicly interact with nonexperts and raise awareness when misinformation is detected; encouraging patients to maintain physical and mental health in ways consistent to COVID-19 regulations; enquiring about and providing support for the concerns patients have on COVID-19; ensuring that the support for COVID-19 concerns on social media is based on an understanding of potential mistrust among users; encouraging the healthy use of social media, as well as the recommendation of social media limitations on the spread on COVID-19 information and lastly the recommendation of reputable sources of COVID-19 related information. The World Health Organization (2020:27–28) also provides a series of five strategies for managing infodemics, which consist of identifying scientific evidence regarding COVID-19; translating knowledge and scientific evidence about COVID-19; reaching out to social media users and communities to amplify action; developing and publishing more scientific evidence through collaboration and developing guidelines on the use of social media for spreading all kinds of information. These strategies will be further discussed in the forthcoming sections of this article.

## Theoretical perspective

**FIGURE 1:**
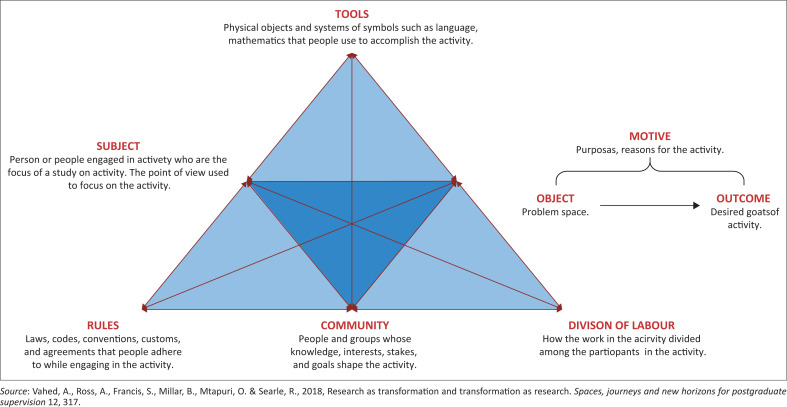
Activity theory.

The chosen theoretical perspective for this study is the Activity Theory, as depicted in Figure 1 (Vahed et al. 2018). Activity Theory was developed by Professor Yrjo Engestrom and served as a method for analysis that bridges the gap between the individual subject and the societal structure (Engestrom, Miettinen & Punamaki 1999). This theoretical model consists of interactive elements that depict interactions using the concept of a collective activity system. The interactive elements of the Activity Theory consist of the tools, subject, object, rules, community and division of labour (Vahed et al. 2018). Regarding the tools, these are the mediated artifacts and resources that can be used for the subject in the activity (Yamagata-Lynch 2010). These may be physical objects or systems that stakeholders can use to accomplish the motive of the activity. The subject refers to all the individuals who engage in the activity, which in this case consists of all the social media users who participate in the infodemic.

On the other hand, the object and rules refer to COVID-19 information shared on social media platforms and relevant conditions that will guide user behaviour on social media, respectively. The community refers to people and groups whose objectives align with the activity, such as the content creators, users, social media policymakers and the public health entities involved. Lastly, the division of labour refers to how the work in the activity will be divided, which, in this study, will consist of the distributed actions and operations relevant to the community (stakeholders) (Vahed et al. 2018).

## Research methodology

**FIGURE 2:**
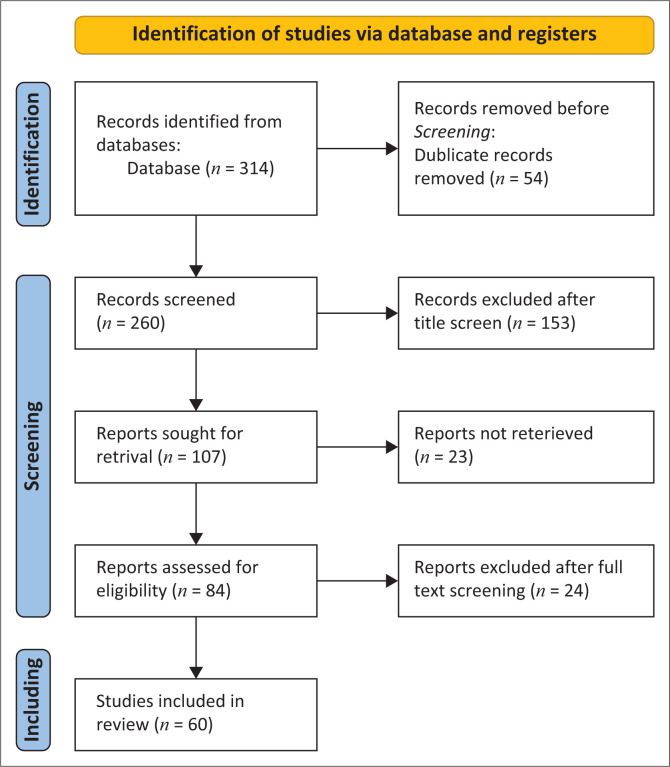
PRISMA model.

The study aims to develop a conceptual framework that can be used to mitigate misinformation about COVID-19 on social media platforms during an infodemic. The methodology used a structured literature review that analysed the nature and characteristics of the research aim. Figure 2 depicts the various steps. The first step was to review related literature to identify the relevant terminology used in the search strategy on various scholarly databases (Web of Science, Scopus, Google Scholar and PubMed). The results were downloaded (*n* = 314) and checked for duplicates (54 duplicate papers) while the titles were screened for relevance, and if inclusion criteria were met, 153 papers were eliminated. The inclusion criteria for this study were original research published after 2019 on infodemics and COVID-19. The next step was to download the remaining 107 studies for full screening. Twenty-three papers could not be retrieved, while 24 were excluded after the full text was screened as these articles did not speak to misinformation or infodemics during the COVID-19 pandemic. Three team members reviewed research papers and identified initial themes for data extraction. Before conducting the study, ethical approval was obtained from the University Research Ethics Committee. The next section of this article presents the study’s findings.

## Results

Sixty articles were identified and included in the data analysis during the structured literature review. These articles were analysed making use of the Activity Theory to understand the different components that would contribute to the object of the framework, which was to mitigate the sharing of misinformation about COVID-19 on social media platforms during an infodemic. The first step during the analysis was to identify the community players and tools that were involved during the COVID-19 pandemic. The community included social media users, healthcare professionals, social media platform regulators, government organisations and other public health entities. The primary tool used to share health information was the social media networks, such as Facebook, Twitter and WhatsApp. However, when considering the issue of who was sharing misinformation about the virus on social media, two distinct subjects were identified during the analysis: firstly the social media users and secondly the social media regulators and policymakers. For each of these subjects, one action, or rule, was identified during the analysis of the articles. Social media users contributed to the increase of misinformation about COVID-19 on social media while the regulators and policymakers were slow to respond to the misinformation on these platforms. The division of labour yielded four distinct themes during the analysis: Identifying evidence; Knowledge translation; Amplifying action; Quantifying impact and Coordination and governance.

## Discussion

**FIGURE 3:**
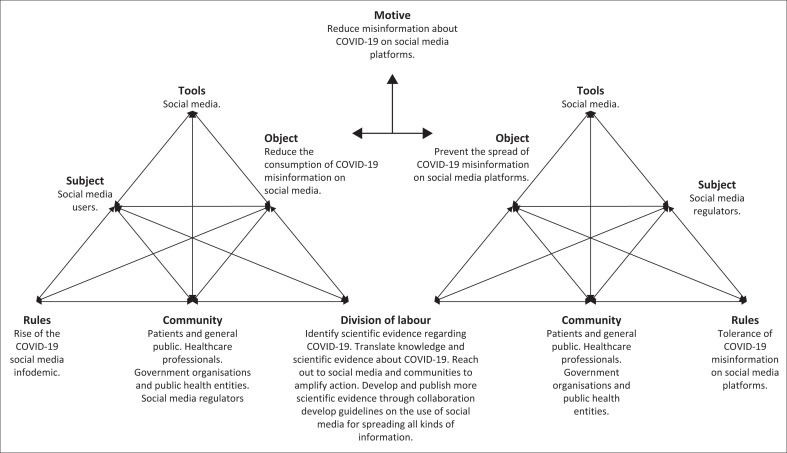
Proposed framework for managing the coronavirus disease 2019 infodemic.

The framework in Figure 3 has been depicted using two interacting activity systems representing the activities set by the two subjects, social media users and regulators. Each of these categories will be discussed in more detail.

### Subject

The subject refers to all the individuals and groups participating in the activity. These people are the overall focus of the study and will be responsible for implementing the strategies identified in the framework (Vahed et al. 2018). The research problem being investigated in the study is focused on social media; thus, the first subject of the study is all the social media users involved in creating, spreading and consuming misinformation on social media platforms (Engestrom 2018). The second subject is the social media regulators and policymakers responsible for managing, regulating and governing social media platforms. In the case of an infodemic, social media regulators and policymakers must govern the spread of misinformation on social media platforms (Nguyen 2021).

### Object

Concerning the object, Yamagata-Lynch (2010:2) states that ‘the object is the goal or motive of the activity’. Given that the purpose of this framework is to mitigate COVID-19 misinformation on social media, the overall motive is to mitigate the sharing of misinformation about COVID-19 on social media platforms. For the first subject, social media users, the object will be to reduce the creation, sharing and consumption of COVID-19 misinformation, while the second is to manage, govern and regulate the spread of COVID-19 misinformation on social media platforms.

### Rules

According to Yamagata-Lynch (2010:2), ‘the rules are any formal or informal regulations that in varying degree can affect how the activity takes place’. The rules in the context of this study are all the codes and customs social media users and regulators follow while they engage on the social media platform. Given that the activities within this framework are relative to the mitigation of COVID-19 misinformation shared on social media platforms, the rules, in this case, are the tolerance of COVID-19 misinformation on social media platforms by platform regulators, as well as the rise of the COVID-19 infodemic on social media facilitated by social media users.

### The community

According to Yamagata-Lynch (2010:2), the community refers to the demographic or social group to which the subject belongs. These are all the stakeholders involved in sharing COVID-19 misinformation on social media platforms during an infodemic. Within this study, the community refers to the social media users of the general public, healthcare professionals, social media platform regulators, government organisations and other public health entities. As it pertains to the patients and general public, these are the various users and content creators participating in spreading COVID-19 misinformation on social media. These individuals play an active role in the ongoing social media infodemic in that they are the ones who actively seek out trusted health information sources on online social media platforms (Grewal et al. 2021). According to Walsh et al. (2021), these individuals often directly interact with health professionals on these platforms when sourcing health-related information and are often responsible for promoting accurate and inaccurate information to their followers. When referring to healthcare professionals, these individuals and organisations create and maintain accurate and contemporary health information on social media platforms.

On the other hand, when referring to social media platforms, not only does this pertain to the online or digital application software, but it also includes the content regulators in charge of creating the social media policies. The social media platforms themselves are responsible for implementing surveillance systems for health misinformation on social media, as well as promoting health information from trusted social media accounts run by health organisations like the World Health Organization and the National Department of Health. Lastly, the government organisations are the mentioned World Health Organizations, the National Department of Health and the Food and Drug Administration, among others. These organisations help improve internet and cellphone access for rural populations and subsidise the creation of high-quality health information on social media (Grewal et al. 2021). These stakeholders are instrumental in sharing and accessing health information on densely populated online social media platforms.

### Tools

The tools are the mediated artifacts and resources that can be used for the subject in the activity (Yamagata-Lynch 2010). These may be physical objects or systems that stakeholders can use to accomplish the motive of the activity (Vahed et al. 2018). The tools used in this activity system are social media platforms and networks. In this study, social media is defined by the researcher as the various websites and media applications that allow its users to create and share content while allowing them to participate in social networking. This framework aims to allocate different strategies among stakeholders to mitigate misinformation about COVID-19 on social media through the division of labour. These strategies will serve as mechanisms for managing COVID-19-related content on these platforms.

### Division of labour

According to Yamagata-Lynch (2010:2), ‘the division of labor refers to how the tasks are shared among the community’. In the conceptual framework, each strategy is to be distributed among each stakeholder to achieve the object and motive of the activity theory. The developed framework utilises five strategies for managing the COVID-19 social media infodemic, which are as follows:

### Identifying evidence

When it comes to identifying evidence, health professionals, governments and other public health entities should contribute to the analysis of social media results and submit their results to the general public. Furthermore, in this strategy, collating, reviewing, appraising and assessing the relevance of information on social media is essential because these activities allow for the formation of recommendations and policies that impact the health of individuals and populations.

### Knowledge translation

This strategy applies to public health entities as it involves translating knowledge and scientific evidence about COVID-19. Effective communication, as well as decisive message delivery by health authorities, is essential in preventing the misrepresentation of information and ensuring that they maintain credibility among social media users. For this to occur, effective translation of health-related knowledge should be facilitated.

### Amplifying action

This strategy applies to healthcare professionals, government organisations, public health entities and social media platform regulators. Amplifying action suggests that governments should reach out to social media users and communities and establish an understanding of what information is needed by people regarding COVID-19. This will encourage active engagement among users and public health professionals, thus creating active communication among these stakeholders and allowing users to elicit information about COVID-19.

### Quantifying impact

This strategy applies to government organisations and healthcare professionals. Quantifying impact involves developing a deeper understanding of COVID-19 by creating a partnership between these two stakeholders. This partnership will facilitate a cross-sectorial and establish international scientific collaboration. Furthermore, this partnership should track trends and the impact of the messages and interventions they facilitate to manage health misinformation on social media effectively.

### Coordination and governance

Lastly, coordination and governance refer to creating international guidelines and policies regarding fake news on social media. This strategy suggests that all stakeholders should be given a set of unified guidelines to help slow down and streamline the flow of information on social media platforms. As pertains to social media regulators, they should specifically be responsible for updating guidelines on fake news, as well as developing a unified strategy and approach to correcting misinformation while producing trusted information in collaboration with governmental organisations and healthcare professionals.

With the necessary tools and strategies for mitigating misinformation being identified, the acquired tasks and labour should then be divided among the stakeholders.

The proposed framework presented in Figure 3 provides a systematic overview of the problem that misinformation about COVID-19 on social media presents. The framework further provides insight into the outcome of the infodemic on social media if it persists during a pandemic, which is likely to cause panic and confusion among the general public resulting in distrust of health care workers and health organisations that are likely to make any efforts to promote the correct health information ineffectively. In some cases, social media users may even put their own health at risk when they pursue ineffective and unsafe information about COVID-19. The framework provides a holistic view of all the role players, tools and activities that are in play during a pandemic. For this purpose, the Activity Theory is an excellent theoretical foundation to systematically identify and analyse all the components that contribute to misinformation about COVID-19 on social media. The framework should be used by government, health organisations and workers to analyse and mitigate the effects of misinformation about the COVID-19 infodemic on social media.

## Recommendations

According to the World Health Organization (2020), the infodemic greatly exacerbates the challenges of the COVID-19 pandemic and impacts citizens of every country. Social media engagement has increased exponentially; thus, any kind of information relative to the COVID-19 virus is likely to spread regardless of the reliability of the source, which then raises concerns regarding the spread of fake news (Cinelli et al. 2020). When managing the COVID-19 infodemic on social media, government needs to consider that the spread of COVID-19 misinformation on social media could likely fuel panic among the general public, which could likely result in the development of negative opinions about healthcare professionals and public health institutions by members of the general public. Furthermore, the evidence collected throughout this study also indicated that COVID-19 misinformation on social media created a distrust of public health institutions by the general public, resulting in some individuals resorting to the pursuit of ineffective and unsafe treatment methods for COVID-19 symptoms.

To manage the spread of COVID-19 information and mitigate the spread of COVID-19 misinformation and disinformation on social media, the study proposes a conceptual framework to mitigate the use of misinformation on social media during COVID-19. The data collected from secondary sources have allowed for the identification and adoption of different strategies that can be used to prevent the spread of misinformation about COVID-19 on social media platforms during the pandemic. These strategies consist of identifying scientific evidence regarding COVID-19, translating the knowledge and scientific evidence collected about COVID-19, reaching out to social media users and communities, developing as well as publishing more scientific evidence through collaboration and developing guidelines on the use of social media for spreading all kinds of information on platforms. The work builds on the research of Wu et al. (2019), which focused on direct detection of misinformation based on the content being shared, the contextual information available on social media, how the misinformation circulates among users and the time in which the information has been available on these platforms. The guidelines can also be used in conjunction with the tools that Safieddine, Dordevic and Pourghomi (2017) identified such as ‘real-time rumour trackers’ that categorise information based on accuracy and inaccuracy, a ‘Node Protector’, which is used by researchers to disseminate correct information, as well as using the algorithmic and practical approaches to evaluate and verify information on social media platforms.

## Conclusion

In light of the identified negative outcomes of the discussed COVID-19 social media infodemic and the spread of misinformation on social media, it can be concluded that there is a need for health information management on social media. This study developed a conceptual framework that can be used to mitigate the sharing of misinformation about COVID-19 on social media platforms during an infodemic. By implementing a set of developed strategies and activities, this framework aims to distribute these activities among stakeholders, to facilitate the management of COVID-19 misinformation on social media. Despite the validity and effectiveness of the findings discovered throughout this study, one major limitation of this research is that the data collected is secondary. For this study to be more effective, gathering primary data should be facilitated in future studies and research in this field. Gathering qualitative data could further facilitate more innovative applications to the developed framework and possibly allow for the framework’s application in other environments outside of social media.
